# What Factors Influenced Young Adults to Vote in the 2020 Presidential Election?

**DOI:** 10.1177/23522798241301440

**Published:** 2024-12-07

**Authors:** Hye-Young Yun, Sandra Graham

**Affiliations:** 1 University of California, Los Angeles, USA

**Keywords:** voting behavior, 2020 presidential election, cross-racial/ethnic friendship, volunteer activity, life value, adolescence, early adulthood

## Abstract

Using data drawn from a racially/ethnically diverse sample of participants (*N* = 1,489; 52% female; M_age_T1_: 18.10; 34% Latino, 21% White, 20% Asian, 11% Black, 11% multiracial/multiethnic, and 3% other), we conducted a binary logistic regression to identify which factors during adolescence and early adulthood were associated with voting behavior in the 2020 presidential election. There were three main findings. First, young adults who had more cross-racial/ethnic friendships and those who participated in volunteer activities during their senior year of high school were more likely to vote. Second, having cross-racial/ethnic friendships and endorsing self-transcendence values (benevolence, universalism) in early adulthood were positively associated with voting behavior, even when controlling for high school factors. Third, those who endorsed self-enhancement values were more likely to vote when they had more cross-racial/ethnic friendships during high school. Implications for voting patterns among young adults in future presidential elections are discussed.

The success of a democratic society relies heavily on the civic and political engagement of its citizens, and one of the primary ways U.S. citizens can participate in civic and political life is by voting (Dewey, 1916; Galston, 2001). In modern presidential elections, the overall voter turnout rate (the proportion of eligible voters who cast a ballot in an election) has fluctuated between approximately 50% and 67% (U.S. Census Bureau, 2014, 2021a). However, young adults (18–24 years old) have voted at consistently lower rates than all other age groups in every presidential election since 1964 (U.S. Census Bureau, 2014, 2021a). For example, in the 2020 election—which saw the highest voter turnout of the 21st century—the turnout among young adults (51.4%) was more than 15 percentage points lower than the overall turnout (66.8%; U.S. Census Bureau, 2021a, b).

Political analysts, campaigns, and researchers have paid a great deal of attention to the voting behavior of young adults in recent election cycles. The Center for Information and Research on Civic Learning and Engagement (CIRCLE, 2020) concluded that voting patterns among young adults had a major impact on the 2020 election. Further, young adult voting behavior has a long-term effect in the sense that voting is a habitual behavior: people who vote three times in a row, in the first three elections for which they are eligible, are more likely to be lifelong voters (Miller et al., 1996; Plutzer, 2002; Verba & Nie, 1972). Therefore, young adult voters are important to the political process not only because they comprise a large portion of the population, but also because they constitute the nation’s future voters. Prompted by the finding of consistently low voter turnout among young adults, researchers have explored the antecedents of voting behavior in this age group. Of the antecedents examined in previous research, the extent of political knowledge or voting information has shown a significant association with voting behavior (Kaid et al., 2000, 2007). Notably, in states where voting officials mailed a 2020 presidential election ballot to every registered voter due to the social distancing restrictions of the COVID-19 pandemic, turnout increased by an average of 5.6% and this increase was even higher among infrequent voters (McGhee et al., 2022). However, approximately 50% of young adults still did not vote. This pattern suggests that a lack of information may not be a major impediment to voting among Generation Z (individuals born after 1995), whose members have had access to a digital infrastructure for communication, organization, and mobilization from a young age (Pew Research Center, 2020a).

A key determinant of political behaviors, which typically begin in early adulthood when people first become eligible to vote, is past politics-related experiences (e.g., Plutzer, 2002). Therefore, late adolescence and the transition to adulthood is likely a critical period for research on what factors affect voting behavior. While prior research has consistently shown that this period matters for shaping political behaviors such as voting later in adulthood (e.g., McFarland & Thomas, 2006; Nie et al., 1996; Putnam, 2000), very few studies have investigated which specific youth and early adulthood experiences independently and simultaneously impact voting behavior. To extend the empirical findings on recent voting behavior among young adults in the United States, we assess which factors from high school and young adulthood influenced their voting behavior in the 2020 presidential election. We focus on the 2020 election for two main reasons. First, the 2020 presidential election saw record turnout among young adult voters (U.S. Census Bureau, 2021a); second, some political observers considered the 2020 presidential election a generational change election and a breakthrough moment for young adult voters (CIRCLE, 2020; U.S. Census Bureau, 2021a). Further, given that most of the growth in the electorate since 2000 has come from non-White (i.e., Asian, Black, Latino) eligible voters (Pew Research Center, 2020b) and the school-aged population is more racially and ethnically diverse than in older generations (National Cental for Education Statistics [NCES], 2024), it is important to explore individual-level racial/ethnic differences as well as other racial/ethnic factors (e.g., exposure to different racial/ethnic contexts, having cross-racial/ethnic friendships) that might contribute to voting behavior.

Accordingly, using a longitudinal sample that includes multiple racial/ethnic groups and moving beyond the focus on demographic differences to also consider developmental factors, the current study had two main goals: First, we address how high school experiences—namely, exposure to racial/ethnic diversity, having cross-racial/ethnic friendships, and being involved in volunteer activities—affect voting behavior in early adulthood. Second, we examine whether experiences in high school and early adulthood are either independently or interactively associated with voting behavior in the 2020 presidential election.

## Gender and Racial/Ethnic Differences

Demographic differences in the pattern of overall voter turnout are historically consistent. Women have registered and voted at higher rates than men in every presidential election since 1980, with the turnout gap between women and men growing slightly larger with each successive presidential election. Concerning racial/ethnic differences, although the turnout rates of Asian, Black, and Latino groups have increased significantly since 2000, Whites still vote at a higher rate than other racial/ethnic groups. While younger adults have tended to vote at lower rates than older adults, the demographic differences within the population of young adults reflect historic patterns among voters overall: young adult women and Whites are more likely to vote than young adult men and members of other racial/ethnic groups (CIRCLE, 2021; U.S. Census Bureau, 2021a).

## Life Values (Self-transcendence and Self-enhancement)

Values, which guide the selection and appraisal of behaviors and events, are considered fundamental predictors of voting behavior (e.g., Schwartz et al., 2010). Schwartz (1992) established a theory of universal patterns in the content and structure of personal values. These universals include a notable pair of contrasting values: self-transcendence and self-enhancement. Researchers have consistently found that both values are associated with a wide range of social behaviors; self-transcendence values focus on care and concern for the welfare of those with whom one has frequent contact (benevolence) or even members of out-groups (universalism), while self-enhancement values focus on self-interest, socially recognized success, and dominance over others (Crompton, 2011; Sagiv et al., 2011; Schwartz et al., 2010; Strauss et al., 2008). Although all people are likely to endorse both self-transcendence and self-enhancement values to some degree (Bardi & Schwartz, 2003), the relative priority people place on each is an important correlate of civic and political behavior. Compared to those who focus on self-enhancement values, people who exhibit a higher level of self-transcendence are less prejudiced, more accepting of diversity, more cooperative and less competitive, and more likely to adopt environmentally sustainable behaviors (Karp, 1996; Sagiv et al., 2011; Strauss et al., 2008). Regardless of political identification, participation in political activities, such as voting, is positively correlated with self-transcendence and negatively correlated with self-enhancement (Schwartz et al., 2010; Vecchione et al., 2015). Specifically, one study found that non-voters tended to value self-enhancement, while voters tended to prioritize self-transcendence (Caprara et al., 2012).

## The Importance of Peers and Exposure to Racial/Ethnic Diversity

Individuals’ perceptions of the values prevalent among those around them can influence their own values, and thus may shape their political attitudes and behaviors, which in turn influence decisions about whether to vote. For example, when individuals believe that others prioritize self-transcendence values, they are more motivated to vote (e.g., Sanderson et al., 2019). Given that interpersonal communication is the most effective channel for the transmission of civic and political information and values among young adult voters (Jasperson & Yun, 2007; Muralidharan & Sung, 2016), political discussions with peers are likely related to increased understanding of and confidence in political matters. One study found that young adult voters who engaged in more face-to-face political discussions were more likely to vote in the 2012 U.S. presidential election (Muralidharan & Sung, 2016). Most previous research finds that among young adults, peer group interactions likely have a significant impact on later voting behavior. Because adolescence is a critical developmental period during which individuals are highly sensitive to the influence of both their peers and the wider society (Blakemore & Mills, 2014), researchers view peer groups at school as an especially important factor for socialization regarding civic engagement (e.g., Algan et al., 2019; Almås et al., 2010; Gradstein & Justman, 2002).

Recent demographic trends have led to dramatic shifts in the racial/ethnic composition of U.S. schools. More than half of school-age youth in the United States are members of racial/ethnic minoritized groups; Latinos are now the largest racial/ethnic minority group in the nation, and Asians are the fastest growing numerical minority group (NCES, 2024; U.S. Census Bureau, 2021c). Although the research on the associations between voting behavior and peer interactions has not kept pace with these changing demographic trends, a few studies have provided results with important implications for the influence of exposure to racial/ethnic diversity and having cross-racial/ethnic friendship on voting behaviors. For example, Polipciuc et al. (2021) found that youth who attended a more racially/ethnically diverse high school had a higher probability of voting in the 2000 presidential elections when they became young adults. This pattern could be explained by increased support for social policies and affirmative action legislation that directly applies to other racial/ethnic groups with which students interact (Boisjoly et al., 2006; Tropp & Pettigrew, 2005). However, given that one of the most effective ways to reduce prejudice is having a cross-racial/ethnic friendship, which go beyond mere exposure to racially/ethnically diverse peers (Davies et al., 2011), school racial/ethnic diversity in and of itself may not necessarily guarantee that a student will have more opportunities to form cross-racial/ethnic friendships. Thus, it is important for researchers to investigate the association between number of cross-racial/ethnic friendships and voting behavior in schools that vary in racial/ethnic diversity.

## Volunteering Activities

In addition to interacting with cross-racial friends, participating in voluntary associations during adolescence and the transition to young adulthood is another type of social interaction that impacts later participation in political activities (e.g., McFarland & Thomas, 2006; Nie et al., 1996; Putnam, 2000). Regardless of whether the association is political or nonpolitical in nature, participating in a voluntary association promotes political activities, including voting (e.g., McFarland & Thomas, 2006; Putnam, 2001; Putnam et al., 1993). Because volunteering is also linked to community engagement, being involved in a voluntary activity broadens a person’s perspective, prompting them to move beyond individualistic concerns and toward promoting feelings of community solidarity, tolerance, and social trust (e.g., Kelly, 2009; Kwak et al., 2004). In turn, young people develop a strong sense of membership in both the voluntary association and the wider community, which motivates them to maintain an interest in political issues. That is, a key impact of volunteering is that youth are educated in ways that facilitate their engagement in political activities in adulthood (e.g., McFarland & Thomas, 2006). Although there is robust evidence that volunteering during adolescence is strongly linked to increased political involvement later (e.g., Jones, 2006; Putnam, 2000; Verba et al., 1995), few studies have explored how volunteer activities in early adulthood affect voting behavior.

## The Current Study

To gain insight into voting behavior during early adulthood, we examined factors associated with voting behavior in the 2020 presidential election, when turnout among young adults was higher than at any other point in the 21st century. Using data drawn from a racially/ethnically diverse sample of participants who were surveyed during their senior year (12th grade) in a California high school and again in the spring of 2021, after they had transitioned out of high school and were eligible to vote in the 2020 presidential election, the current study addressed two primary research goals. First, we examined how high school experiences (number of cross-racial/ethnic friendships, school racial/ethnic diversity, involvement in volunteer activities) were associated with later voting behavior, accounting for gender and race/ethnicity. Based on the findings of prior studies as well as the associations between these three high school factors and taking diverse perspectives, awareness of social justice, and the development of diverse social networks (e.g., Camino & Zeldin, 2002; Crystal & DeBell, 2002; Hart et al., 2007; Youniss & Yates, 1997), we hypothesized that participants who had more cross-racial/ethnic friendships, had more exposure to racially/ethnically diverse school contexts, and were more involved in volunteer activities during high school would be more likely to vote in the 2020 presidential election.

Next, we investigated whether high school and young adulthood factors (number of cross-racial/ethnic friendships, involvement in volunteer activities, life values) were linked either independently or interactively to voting behavior in the 2020 presidential election. Because few studies have considered both high school and early adulthood, we did not develop specific hypotheses for this step of the analysis. However, given that adolescence is a particularly important developmental period for the definition of an individual’s identity as a member of society, and this identity is strongly related to civic or political behaviors (e.g., Erikson, 1968), we assumed that the association between high school experiences (having cross-racial/ethnic friendships, involvement in volunteer activities) and voting behavior would remain strong even after accounting for early adulthood experiences. Further, life values, which underlie most attitudes, lend coherence to core political values and guide people’s actions (Rokeach, 1973; Schwartz, 1992; Schwartz et al., 2010); thus, we speculated that young adults who regarded self-transcendence values (benevolence, universalism) as more important than self-enhancement values might be more likely to engage in voting as a political action to address issues of social inequality.

## Method

### Participants

The sample used in the analyses (*N* = 1,489, 52% female) includes participants who completed two waves of a longitudinal study (UCLA Middle School & High School Project), the first during the spring of their senior year (12th grade) in a California high school and the second in the spring of 2021 after they had transitioned out of high school, and who were eligible to vote in the 2020 presidential election. Based on self-reports, the racial/ethnic composition of the baseline sample used in the analyses was 34% Latino, 21% White, 20% Asian, 11% Black, and 11% multiracial/ethnic. The remaining 3% of participants identified as American Indian, Middle Eastern, or Other; these groups were collapsed into the “other” category because they were too small for separate analysis.

### Procedure

The original sample (*N* = 5,991, 52% female) was recruited from 26 racially/ethnically diverse middle schools in California over a span of three consecutive years. The survey was approved by the Institutional Review Board at the University of California, Los Angeles. For the original survey, each cohort was surveyed annually starting when they were in sixth grade in 2009, 2010, or 2011 (Wave 1 = W1) and continuing after their senior year in high school. The pre-graduation data (12th grade, Wave 8 = W8) used as a baseline for the current analyses were collected from the three cohorts in the spring of their senior year in high school (2015, 2016, or 2017). The first survey after students had graduated from high school was conducted in 2016, 2017, and 2018 for the three cohorts (Wave 9 = W9).

In the fall of 2020, the survey team contacted all 3,080 participants from the previous wave (W9, the first survey after students had graduated from high school) to confirm or update their contact information. Responses were received from 2,002 individuals, all of whom were subsequently contacted for recruitment into the current study. Of those who were invited to participate, 78% (*N = *1,557) completed the survey in the spring and early summer of 2021 (Wave 10 = W10). All surveys were completed online; participants received a weblink to the survey platform via email. The surveys took 50 to 60 min to complete; respondents received $50 for completing the survey. The timespan between the respondent’s senior year in high school (W8) and W10 in 2021 varies by cohort, ranging from 2 to 4 years. At W10, participants ranged from 20 to 24 years old (*M = *21.72, *SD = *0.81), and the mean age difference between consecutive cohorts was approximately 1 year (cohort 1: *M = *22.46 years, *SD = *0.52; cohort 2: *M = *21.46 years, *SD = *0.53; cohort 3: *M = *20.52 years, *SD = *0.54).

### Measures

#### Cross-racial/Ethnic Friendships

During the spring of 12th grade (W8) and again 2 to 4 years after their transition out of high school (W10), participants were asked to list the names of their friends via a peer nomination procedure. Participants also reported whether each friend was “the same ethnic group as me.” Responses were coded such that same-race/ethnicity friends (0) served as a comparison for cross-racial/ethnic friends (1). Number of cross-racial/ethnic friendships was measured via a summed score, with higher scores indicating more cross-racial/ethnic friendships (W8: range = 1–7, *M = *6.50, *SD = *3.34; W10: range = 1–7, *M = *5.95, *SD = *3.50).

#### Volunteer Activities

Four items, which were adapted from the Active and Engaged Citizenship (AEC) questionnaire (Bobek et al., 2009), were used to assess volunteer activities (e.g., “Volunteered your time to help people in your community,” “Volunteered for an environmental group to recycle or stop pollution,” “Volunteered for a group that worked to reduce prejudice”). During the spring of 12th grade (W8) and again 2 to 4 years later (W10), participants reported the frequency of their volunteer activities over the past year on a 5-point scale ranging from 1 (*never*) to 5 (*more than once a month*). A higher average score on these four items represents more frequent engagement in volunteer activities (W8: *M = *1.81, *SD = *0.78, α = .72; W10: *M = *1.43, *SD = *0.71, α = .76).

#### High School Racial/Ethnic Diversity

To measure racial/ethnic diversity in respondents’ high schools, we used school-level race/ethnicity data collected from the California Department of Education (CDE) to calculate Simpson’s (1949) diversity index:

$$D_{S}=1-\sum_{i-1}^{g}p_{i}^{2}$$

where p is the proportion of students in the school who are in racial/ethnic group *i*. This proportion is squared ( $p_{i}^{2}$ ), summed across *g* groups, and then subtracted from 1. *D_S_* gives the probability that any two students randomly selected from a school will be from different racial/ethnic groups. Values range from 0 to approximately 1, with higher values indicating greater diversity (i.e., there are more racial/ethnic groups and they are represented relatively evenly with no clear numerical majority). Thus, Simpson’s index captures both the number of distinct groups in a setting and the relative size of each group.

Because students transitioned from 26 middle schools to approximately 200 high schools in 9th grade, the number of respondents in a given school in 12th grade varied widely (range: 1–128) and most high schools did not have enough individuals in our sample to warrant multilevel analysis (e.g., Maas & Hox, 2005; McNeish & Stapleton, 2016); of the 200 schools, 104 included only one student. Thus, school racial/ethnic diversity was considered at the individual level as a measure of a student’s exposure to racial/ethnic diversity at school. *D_S_* ranged from 0.03 to 1.00 (*M = *0.61, *SD = *0.14), indicating moderate to high diversity.

#### Life Values: Self-transcendence Versus Self-enhancement in Young Adulthood

To examine participants’ life values, the survey asked, “When you think about your life, how important is each of the following to you, personally?” Participants answered this question for each of 10 items on a 5-point scale ranging from 1 (*not at all important*) to 5 (*very important*) (Furco et al., 1998). A principal-components analysis with varimax rotation was conducted on the responses. Inspection of the eigenvalues, scree plot, and factor loadings showed that three factors underlie these 10 items. The first factor, referred to hereafter as *self-transcendence*, includes five items that tap the degree to which the respondent is concerned with the welfare and interests of their community and other people more generally (e.g., “Helping my community,” “Working to stop prejudice,” “Helping people who are less fortunate”). The second factor, referred to hereafter as *self-enhancement*, consists of three items that tap the degree to which the respondent values personal success and dominance over others (e.g., “Making a lot of money,” “Having a stable/well-paying job,” “Living in a big house”). The third factor consists of two items that assess the importance of patriotic values (“Serving my country” and “Helping my country”). The current study focuses on the first two factors, preference for self-transcendence (*M = *4.07, *SD = *0.85, α = .90) and preference for self-enhancement (*M = *3.66, *SD = *0.86, α = .70).

#### Voting Behavior

The outcome variable is a binary measure of voting behavior in the 2020 presidential election. Participants were asked “Did you vote in the 2020 presidential election?” and could answer either *yes* (=1) or *no* (=0).

### Analytic Plan

The study proceeded in two steps. First, we calculated descriptive statistics and correlations to estimate the relations between the hypothesized predictors. Second, we conducted three binary logistic regression models via the IBM Statistical Package for the Social Sciences (SPSS version 22) to examine the associations between the predictors and voting behavior. Model 1 investigates how high school experiences (number of cross-racial/ethnic friendships, school racial/ethnic diversity, involvement in volunteer activities) were associated with later voting behavior, accounting for gender and race/ethnicity. Model 2 examines whether the same three high school factors included in Model 1 and the young adulthood factors (number of cross-racial/ethnic friendships, involvement in volunteer activities, life values) were linked independently to voting behavior in the 2020 presidential election. Model 3 includes all variables in Model 2 and adds two-way interactions between the high school and young adulthood experiences. Because the original survey was fielded among three cohorts (starting in 2009, 2010, or 2011; W1), dummy variables for cohort membership were included as control variables in all models. Missing data were handled via listwise deletion according to the SPSS protocol.

## Results

### Descriptive Analyses

In the analytical sample, 85% of participants voted in the 2020 presidential election and 15% did not. Electoral participation among respondents differed by gender and race/ethnicity. Consistent with Census data, women voted at a much higher rate (69%) than men (31%). Regarding racial/ethnic differences, Whites had the highest rate of voting in the 2020 election (94%) and Asians had the lowest rate (79%). Multiracial, Black, and Latino respondents fell in the middle, with rates of 90%, 84%, and 82%, respectively. The overall gender difference in voting behaviors persisted within racial/ethnic groups: women were more likely to vote than men within each group (voting percentages by gender were as follows: Asians: 84% of women and 67.5% of men, Blacks: 86% and 78%, Latinos: 87% and 71%, Whites 93% and 90%, multiracial 90% and 82%).

As hypothesized, participants’ high school experiences were correlated with their voting behavior (see Table 1); specifically, having more friends from different racial/ethnic groups in high school ( γ = .13, *p < *.001), volunteering more frequently ( γ = .11, *p < *.001), and being exposed to more racial/ethnic diversity during high school ( γ = .08, *p < *.01) were positively correlated with voting in 2020. Further, having more cross-racial/ethnic friendships in early adulthood was associated with voting in the 2020 presidential election ( γ = .16, *p < *.001). In addition, endorsing self-transcendence values during early adulthood was significantly positively associated with voting behavior ( γ = .23, *p < *.001) but endorsing self-enhancement values was not. Finally, consistent with the patterns for high school experiences, volunteering frequently during early adulthood was significantly positively correlated with voting in the 2020 presidential election ( γ = .09, *p < *.001).

**Table 1.** Correlations, Means, and Standard Deviations of Study Variables.

Variables	1	2	3	4	5	6	7	8
1. Voting behavior	1							
2. Cross-racial/ethnic friendships at W8	.13***	1						
3. School racial/ethnic diversity at W8	.08**	.08***	1					
4. Volunteer activity at W8	.11***	.07***	.03	1				
5. Cross-racial/ethnic friendships at W10	.16***	.41***	.12***	.07*	1			
6. Self-transcendence at W10	.23***	.08**	.03	.17***	.06*	1		
7. Self-enhancement at W10	−.05	−.08**	−.10***	−.04	−.13***	.15***	1	
8. Volunteer activity at W10	.09***	.04	.09**	.28***	.04	.22***	−.07**	1
Mean	.85	6.50	.61	1.81	5.95	4.07	3.66	1.43
Standard deviation	.36	3.34	.14	.78	3.50	.85	.86	.71

*Note.* W8 = 12th grade, W10 = early adulthood.

* *p* < .05. **p < .01. ***p < .001.

There were also significant associations between non-voting variables. Exposure to racial/ethnic diversity during high school was significantly positively correlated with the number of cross-racial/ethnic friendships both during high school ( γ = .08, *p < *.001) and after high school ( γ = .12, *p < *.001) and with involvement in volunteer activities in early adulthood ( γ = .09, *p < *.01). Participants who reported more cross-racial/ethnic friendships both during and after high school tended to score higher on self-transcendence (W8: γ = .08, *p < *.01; W10: γ = .06, *p < *.01). Endorsing self-transcendence values was significantly positively associated with endorsing self-enhancement values ( γ = .15, *p < *.001). However, self-enhancement was negatively correlated with cross-racial/ethnic friendship both during and after high school (W8: γ = −.08, *p < *.01; W10: γ = −.13, *p < *.001), as well as with high school diversity ( γ = −.10, *p < *.001). Further, self-enhancement was not related to volunteer activity in high school but was negatively related to volunteer activity after high school ( γ = −.07, *p < *.01). In contrast, endorsing self-transcendence values was significantly positively associated with volunteer activity both during and after high school (W8: γ = .17, *p < *.001; W10: γ = .22, *p < *.001). Participants who had more cross racial/ethnic friendships in high school participated in significantly more volunteer activities in high school ( γ = .07, *p < *.001), however, there was not a significant correlation between attending a more racially/ethnically diverse school and involvement in volunteer activities in high school. Finally, being involved in volunteer activities in high school was significantly positively correlated with volunteering in early adulthood ( γ = .28, *p < *.001).

### Binary Logistic Regression Results

A binary logistic regression was conducted to determine the combined effects of the predictors on the likelihood that a respondent voted in the 2020 presidential election. The final model was statistically significantly ( χ ^2^(16) = 149.91, *p* < .001) better than the null model, explaining 26% (Nagelkerke *R*^2^) of the variance in voting behavior and correctly classifying 87% of cases.

When only high school experience variables were included in the model (see Table 2), for a one-unit increase in the number of cross-racial/ethnic friendships, there was a .15 increase in the log-odds of voting, holding all other independent variables constant (*OR* = 1.16, 95% CI [1.09, 1.23]), while a one-unit increase in volunteer activity increased the log-odds of voting by .46 (*OR* = 1.58, 95% CI [1.20, 2.08]). In addition, women were more likely to vote than men (*OR* = 2.25, 95% CI [1.55, 3.26]) and White respondents were more likely to vote than those in any other racial/ethnic group (Asian: *OR* = .19, 95% CI [.10, .35]; Black: *OR* = .30, 95% CI [.14, .65]; Latino: *OR* = .37, 95% CI [.20, .68]; other: *OR* = .33, 95% CI [.12, .88]).

**Table 2.** Binary Logistic Regression Model1 Predicting Voting Behavior.

Variables	β	*SE*	*Wald*	*df*	*OR* [95% CI]
Gender	.81	.19	18.42	1	2.25*** [1.55, 3.26]
Asian	−1.68	.31	28.55	1	.19*** [.10, .35]
Black	−1.20	.40	9.17	1	.30** [.14, 0.65]
Latino	−1.01	.32	10.14	1	.37** [.20, .68]
Multiracial/ethnic	−.77	.45	2.95	1	.46 [.19, 1.12]
Other	−1.11	.50	4.92	1	.33* [.12, .88]
Cross-racial/ethnic friendships at W8	.15	.03	23.70	1	1.16*** [1.09, 1.23]
High school diversity	1.23	.67	3.38	1	3.41 [.92, 12.59]
Volunteer activity at W8	.46	.14	10.37	1	1.58** [1.20, 2.08]
Cohort 1	−.08	.37	.05	1	.92 [.45, 1.90]
Cohort 2	.09	.37	.06	1	1.10 [.53, 2.27]

*Note.* W8 = 12th grade, W10 = early adulthood, Gender: male = 0; female = 1. Ethnicity: White is the reference group. Cohort: Cohort 3 is the reference group. *SE* = standard error; *df* = degrees of freedom; *OR* = odd ratio; CI = confidence interval.

* *p* < .05. **p < .01. ***p < .001.

When we added early adulthood (post-high school) variables to the model (see Table 3), the associations between voting behavior and the demographic and high school experience variables remained quite stable. Women tended to vote more than men (*OR* = 1.85, 95% CI [1.22, 2.79]) and White respondents remained significantly more likely to vote than those in any other racial/ethnic group. However, while there was no difference in voting behavior between White respondents and multiracial/ethnic respondents when considering only the high school factors, multiracial/ethnic respondents were less likely to vote than Whites when considering both high school and early adulthood factors (*OR* = .24, 95% CI [.09, .63]). For life values, a one-unit increase in self-transcendence increased the log-odds of voting in the 2020 presidential election by .68 (*OR* = 1.98, 95% CI [1.56, 2.50]) but self-enhancement was not significantly associated with voting behavior. Further, in line with the results for high school experiences (*OR* = 1.09, 95% CI [1.02, 1.17]), a one-unit increase in the number of cross-racial/ethnic friendships during young adulthood increased the log-odds of voting by .13 (*OR* = 1.14, 95% CI [1.07, 1.22]). In contrast, involvement in volunteer activities in early adulthood was not significantly associated with voting behavior when high school factors were controlled, but volunteer activities during high school remained significantly associated with voting behavior even when other high school factors were held constant (*OR* = 1.33, 95% CI [1.00, 1.77]).

**Table 3.** Binary Logistic Regression Model2 Predicting Voting Behavior.

Variables	β	*SE*	*Wald*	*df*	*OR* [95% CI]
Gender	.61	.21	8.45	1	1.85** [1.22, 2.79]
Asian	−1.87	.37	26.20	1	.15*** [.08, .32]
Black	−1.59	.45	12.51	1	.20*** [.08, .49]
Latino	−1.36	.37	13.67	1	.26*** [.13, .53]
Multiracial/ethnic	−1.42	.49	8.43	1	.24** [.09, .63]
Other	−1.62	.54	8.96	1	.20** [.07, .57]
Cross-racial/ethnic friendships at W8	.09	.04	6.04	1	1.09* [1.02, 1.17]
High school diversity	.65	.73	.80	1	1.92 [.46, 8.05]
Volunteer activity at W8	.29	.15	3.92	1	1.33* [1.00, 1.77]
Cross-racial/ethnic friendships at W10	.13	.04	14.56	1	1.14*** [1.07, 1.22]
Volunteer activity at W10	.17	.19	.85	1	1.19 [.82, 1.71]
Self-transcendence at W10	.68	.12	32.38	1	1.98*** [1.56, 2.50]
Self-enhancement at W10	−.16	.12	1.69	1	.85 [.67, 1.09]
Cohort 1	−.01	.39	.00	1	.99 [.47, 2.12]
Cohort 2	.09	.39	.05	1	1.09 [.51, 2.35]

*Note.* W8 = 12th grade, W10 = early adulthood, Gender: male = 0; female = 1. Ethnicity: White is the reference group. Cohort: Cohort 3 is the reference group. *SE* = standard error; *df* = degrees of freedom; *OR* = odd ratio; CI = confidence interval.

* *p* < .05. **p < .01. ***p < .001.

**Figure 1.**
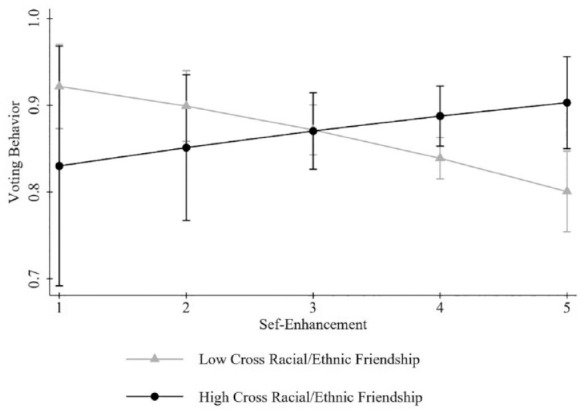
Moderating effect of cross-racial/ethnic friendship on the association between self-enhancement value and voting behavior.

Lastly, as shown in Table 4, the results of the final model revealed an interaction effect between number of cross-racial/ethnic friendships during high school and the value placed on self-enhancement after high school (see Figure 1). Specifically, for a one-unit increase in self-enhancement combined with a one-unit increase in number of cross-racial/ethnic friendships in high school, there was a .07 increase in the log-odds of voting in the 2020 presidential election (*OR* = 1.08, 95% CI [1.00, 1.16]).

**Table 4.** Binary Logistic Regression Model3 Predicting Voting Behavior.

Variables	β	*SE*	*Wald*	*df*	*OR* [95% CI]
Gender	.63	.21	8.81	1	1.88** [1.24, 2.85]
Asian	−1.88	.37	26.06	1	.15*** [.08, .32]
Black	−1.57	.45	12.05	1	.21*** [.09, .51]
Latino	−1.36	.37	13.52	1	.26*** [.13, .53]
Multiracial/ethnic	−1.42	.49	8.36	1	.24** [.09, .63]
Other	−1.60	.54	8.72	1	.20** [.07, .58]
Cross-racial/ethnic friendships at W8	−.19	.14	1.97	1	.83 [.63, 1.08]
High school diversity	.72	.74	.94	1	2.05 [.48, 8.70]
Volunteer activity at W8	.28	.15	3.33	1	1.32* [1.00, 1.75]
Cross-racial/ethnic friendships at W10	.13	.04	14.43	1	1.14*** [1.07, 1.22]
Volunteer activity at W10	.17	.19	.81	1	1.18 [.82, 1.7]
Self-transcendence at W10	.71	.12	33.61	1	2.03*** [1.60, 2.57]
Self-enhancement at W10	−.60	.25	5.85	1	.55* [.34, .89]
Cross-racial/ethnic friendships at W8 × self-enhancement at W10	.07	.04	4.28	1	1.08* [1.00, 1.16]
Cohort 1	.08	.39	.04	1	1.09 [.50, 2.34]
Cohort 2	−.03	.39	.01	1	.97 [.45, 2.08]

*Note.* W8 = 12th grade, W10 = early adulthood, Gender: male = 0; female = 1. Ethnicity: White is the reference group. Cohort: Cohort 3 is the reference group. *SE* = standard error; *df* = degrees of freedom; *OR* = odd ratio; CI = confidence interval.

* *p* < .05. **p < .01. ***p < .001.

## Discussion

According to a recently released report on the 2022 midterm elections, more than 8 million young adults who had turned 18 years old since the 2020 presidential election were eligible to vote in a federal election for the first time (CIRCLE, 2022). Further, these newly eligible voters were notably more racially/ethnically diverse than the rest of the electorate (CIRCLE, 2022). Looking to the near future, the U.S. Census Bureau has predicted that Gen Z (age 9–24 in 2021) and millennials (age 25–40 in 2021) will constitute a majority of potential voters by 2028 and over 60% of potential voters by 2036 (U.S. Census Bureau, 2021d). Given this ongoing shift in both the generational and racial/ethnic distribution of the electorate, it is important to identify the factors associated with engagement in voting behavior using data from a racially/ethnically diverse sample of adolescents and young adults. Accordingly, the current study examined the combined influence of high school and young adulthood experiences on voting behavior in the 2020 presidential election. The results make two significant contributions to the extant research on the voting behavior of young adults. First, we identified the longitudinal effects of high school experiences on voting behavior in the 2020 presidential election. Second, we assessed whether experiences in high school and young adulthood were associated with voting behavior independently and we tested whether high school and young adulthood factors exhibited an additive or interaction effect on voting behavior.

The results regarding gender and racial/ethnic differences were consistent with previous studies and national reports (e.g., CIRCLE, 2021; U.S. Census Bureau, 2021a, b). Women and Whites were more likely to vote in the 2020 election than men and members of racial/ethnic minority groups (Asian, Black, Latino). Of the focal racial/ethnic minority groups in the current study, the multiracial group had the highest turnout and Asian respondents had the lowest turnout.

In the models of high school experiences, having more cross-racial/ethnic friendships and being involved in more volunteer activities in 12th grade were associated with an increased likelihood of voting in the 2020 presidential election. Prior research has proposed that having cross-racial/ethnic friendships is an important source of knowledge and improves an individual’s ability to see the world from the perspective of others (e.g., Korgen, 2002; Plummer et al., 2016), which promotes the development of a critical consciousness, for example, becoming aware of racial/ethnic inequalities (e.g., Diemer & Li, 2011; Ku et al., 2015; Todd & Galinsky, 2014; Todd et al., 2012). Notably, given that peers influence the links between critical consciousness, perceived capacity to change social conditions, and participation in political action (e.g., Diemer & Li, 2011; Ginwright & James, 2002), having more cross-racial/ethnic friendships might increase involvement in political actions (voting behavior) by helping adolescents take the perspective of racially/ethnically diverse peers when considering social issues.

The relation between school diversity and voting behavior in the current study was more complex than described by Polipciuc et al. (2021) in an earlier study. Specifically, we found that attending a racially/ethnically diverse high school was not associated with voting behavior, while there was a positive correlation between attendance at a diverse school and voting behavior. This pattern suggests that exposure to a racially/ethnically diverse school context does not guarantee a student will have cross-racial/ethnic friendships. The reason for these divergent friendship experiences in diverse schools might be twofold: adolescents’ preference for same-race/ethnicity friendships (e.g., Thijs & Verkyten, 2014) and the use of a subject-based curriculum in high school. Specifically, the widespread practice of academic tracking particularly in STEM courses limits opportunities to form relationships with different-race peers (Moody, 2001). Further, Ragins and Ehrhardt (2021) found that among students who participated in a racial/ethnic diversity training at school, only those who formed cross-racial/ethnic friendships exhibited improved perspective-taking abilities. Accordingly, having cross-racial/ethnic friendships, rather than mere exposure to racial/ethnic diversity at school, likely prompts the development of diverse perspective-taking and critical consciousness, which may lead to more active participation in elections. This finding suggests that researchers should not conceptualize school racial/ethnic diversity as a structural variable that remains stable within a school, but rather approach diversity as a dynamic variable, capturing the everyday experiences of students and the degree to which they experience genuine opportunities to form cross-racial/ethnic friendships (Graham, 2018).

In line with previous research, the current study also found that participating in volunteer activities in high school was associated with voting behavior in young adulthood. Engaging in volunteer activities in the community might spur adolescents to connect the abstract concepts and social issues addressed in the classroom to tangible real-world scenarios that occur in various social networks (e.g., Crystal & DeBell, 2002; Youniss & Yates, 1997), and thus prompt them to focus on community concerns and realize the importance of their rights as contributing members of their community and country (e.g., Watts & Flanagan, 2007). Adolescents likely develop a civic or political identity as well as a sense of connectedness to the community via volunteer activities, and these civic and political interests and attitudes can persist into early adulthood (for a review, see McFarland & Thomas, 2006), possibly providing an impetus to vote.

Turning to the results for the simultaneous assessment of high school and early adulthood factors, having cross-racial/ethnic friendships and being involved in volunteer activities during high school remained significantly associated with voting behavior when early adulthood factors (cross-racial/ethnic friendships, volunteer activities, life values) were considered. However, while having cross-racial/ethnic friendships during early adulthood was also associated with voting behavior in the full model, engaging in volunteer activities during early adulthood was not. Previous studies suggested that participating in volunteer activities in the community during adolescence fosters civic identity formation and provides youth with opportunities to develop both a sense of contributing to society and confidence that their actions are worthwhile (e.g., Camino & Zeldin, 2002; Hart et al., 2007; Lakin & Mahoney, 2006), which together generate a set of values that guide future civic or political engagement (Sherrod et al., 2005). Because adolescence is a critical time for the development and crystallization of civic or political identity and attitudes (e.g., Erikson, 1968), high school experience might have a stronger impact on voting than early adulthood experience. Moreover, the current results might have been driven by participants’ greater geographical stability in high school than post-high school (Arnett, 2000), allowing high school students to exhibit a greater sense of rootedness in their communities through volunteerism. Future studies should examine the reasons for the developmental difference in the effect of volunteering on voting behavior between adolescence and early adulthood that was identified in these results.

In addition to social interaction factors, endorsing self-transcendence values (e.g., working to stop prejudice, helping people who are less fortunate, helping people of different ethnic groups get along better) in young adulthood was positively associated with voting behavior in the 2020 presidential election, while endorsing self-enhancement values (e.g., making a lot of money, having a stable or well-paying job, living in a big house) was not. These findings are consistent with the results of previous studies (e.g., Schwartz et al., 2010; Vecchione et al., 2015). According to the economic theory of democracy (Downs, 1957), decisions about whether to vote are based on costs and benefits; potential voters consider the time and effort that preparing to vote and actually voting would require (costs) and the extent to which they prefer one candidate over the others (benefits). For example, the more potential voters care about who wins, they are more likely to vote. Individuals who endorse self-transcendence values (benevolence or universalism) are more concerned about social justice, and thus have a strong preference for who wins the election because the outcome could influence social inequality. In contrast, individuals who endorse self-enhancement values (self-success) may not be as invested in who wins, and thus are less likely to expend the time and effort required to vote.

Endorsing self-enhancement values did not have a main effect on voting behavior; however, among those who endorsed self-enhancement values, having more cross-racial/ethnic friendships in high school was positively associated with voting. As shown in the results, endorsing self-transcendence values and endorsing self-enhancement values were positively correlated—people are likely to endorse both types of values to some degree but prioritize one or the other (Bardi & Schwartz, 2003). Given that voters who engaged in more face-to-face political discussions with friends before the 2012 U.S. presidential election were more likely to vote in that election (e.g., Huckfeldt, 2021; Muralidharan & Sung, 2016), it is likely that having political discussions with peers from diverse backgrounds in high school serves as an effective channel for the transmission of election information and political action. Cross-racial/ethnic friendships formed in high school might motivate voting behavior more than those formed in adulthood because of the strong benefits of the long-term maintenance of friendships (e.g., Lessard & Juvonen, 2022). Measurement at only two time points prevented us from reaching a clear conclusion about whether cross-racial/ethnic friendships formed in high school were maintained through early adulthood. Future studies should explore how the quality and length of cross-racial/ethnic friendships are related to voting behavior.

### Limitations and Future Directions

Although the present study has many strengths, it also has important limitations, especially regarding generalization and measurement issues. First, readers should take caution when generalizing the findings because of the high 2020 voter turnout rate (85%) in the sample—this rate with a California sample is higher than the 2020 nationwide turnout rate (around 50%–55%) and even higher than the nationwide turnout rate among college students (66%; Institute for Democracy and Higher Education, 2021). Generally, characteristics such as a high level of education or stronger interest in and motivation to participate in research are strong predictors of retention in longitudinal surveys (e.g., Cooley et al., 2003; Gul & Ali, 2010). Notably, among the participants who were surveyed during their senior year in a California high school and in the spring of 2021 in the current study, about 70% remained in the UCLA Middle School & High School longitudinal study for the entire 10-year period. Continuing to participate in the study after graduating from high school might be associated with a strong tendency to engage in political action. Thus, there may be selection bias in this sample. Nevertheless, the racial/ethnic distribution of the focal sample is similar to that of the young adult U.S. population (Latinos are now the largest numerical racial/ethnic minority group in the nation; NCES, 2024; U.S. Census Bureau, 2021c). Further, the findings regarding gender and racial/ethnic differences (Whites versus non-Whites) in voting behavior in the 2020 presidential election are consistent with the results of previous reports using nationally representative data (U.S. Census Bureau, 2021a, b), which suggests that the findings reported herein have implications for voting patterns in future presidential elections.

A second limitation is that the data were collected in California, the largest and one of the most racially/ethnically diverse and solidly Democratic states in the nation. Although we did not study voting preferences in this study, it is likely that the majority of our young adult sample voted for the Democrat Joe Biden. It could be that exposure to diversity and cross-race/ethnic friendships only predict voting behavior of young adults with a more liberal political orientation, thus limiting the generalizability of the findings. Future research will need to investigate whether the context factors examined here predict voting behavior of emerging adults in contexts that vary in geography, urbanicity, political persuasions, and racial/ethnic diversity.

Because the data were limited to high school and early adulthood, a third limitation is that the results cannot be used to draw conclusions about the most critical period for promoting voting behavior or whether high school factors affect long-term voting behavior. Research shows that elementary and middle school students in democratic countries are able to understand the role of government and develop trust in government-related institutions. Even before eighth grade, students’ patterns of political attitudes already match those of adults in their society in many respects (Torney-Purta & Lopez, 2006). Hence, the elementary or middle school years might be just as important as the high school years for facilitating voting behavior in early adulthood. Future studies should use a longitudinal design that analyzes data from earlier school years (elementary and middle school) to identify factors that influence later voting behaviors.

A fourth limitation is related to the specific time the study was conducted. According to recent reports (e.g., Picchio & Santolini, 2022), the record-high voter turnout among young adults in the 2020 U.S. presidential election might have been a temporary effect of the COVID-19 pandemic. At the end of November 2020, the extremely high rate of COVID-19 cases and deaths in the United States and growing social polarization increased motivation to go to the polls among young adults, who tended to favor the Democratic candidate Joe Biden over Republican incumbent Donald Trump (e.g., Baccini et al., 2021). Thus, researchers should examine whether the current findings resulted from a temporary COVID-19 boost or whether the factors identified in this study continue to influence voting behavior after early adulthood.

### Implications for Social Studies Education With an Emphasis on Diversity Values

Researchers and policymakers have emphasized that young adults’ voting behavior is important because individuals who vote in early adulthood are more likely to be lifelong voters (Miller et al., 1996; Plutzer, 2002; Verba & Nie, 1972). Considering that the first three elections a person participates in can significantly shape their voting behavior over the long term, the recent low turnout among young adults may predict poor turnout in future elections. To address low turnout among young adults, high school students should be encouraged to preregister to vote before they reach the legal voting age; however, inconsistent implementation across states, districts, and schools would hinder the effectiveness of this strategy (National Conference of State Legislatures, 2024). Thus, mandating that all high schools offer students preregistration opportunities and voting “run-throughs” for upcoming elections would likely foster political behavior among young adults by establishing voting as a normative behavior from a young age. While mailing a ballot to every registered voter could potentially boost turnout, as seen in the 2020 presidential election (McGhee et al., 2022), the fact that almost 50% of young adults did not vote indicates the need to explore factors beyond simply conveying information before an election.

The current findings imply that high school experiences matter for voting behavior in early adulthood. In particular, fostering cross-racial/ethnic friendships in school and encouraging students to engage in volunteer activities outside school may be unique ways to shape young adults’ politics-related values and help them recognize the significance of their participation in political behavior. Because high school students typically dedicate a significant amount of their daily time to school-related activities (Education Commission of the States, 2023), school context can help develop informed and engaged citizens who are equipped to participate meaningfully in their communities and the political process. Specifically, schools can do this by implementing a social studies curriculum, including classes such as history, geography, civics, economics, and sociology, which collectively provide students with a comprehensive understanding of society and the electoral system (Rodrigez, 2020).

Journell et al. (2015) emphasized that incorporating a disciplinary approach to understanding polling data and political behavior in social studies courses helps students think critically and politically about the real world around them. For Generation Z—whose members are digital natives and a substantial portion of the electorate for future elections—it is particularly important to enhance their media literacy (the ability to critically assess and evaluate media messages) so they can effectively navigate the vast amount of political information available and discern between credible and unreliable sources, allowing them to make informed voting decisions and resist the influence of misinformation. It is also critical that contemporary social studies curricula move away from the master narratives that emphasize patriotism and deference to the White majority to better celebrate the experience of racial/ethnic minority groups in this country, both historically and in the present (Rodrigez, 2020). Leveraging the social studies curriculum by incorporating materials on current political issues and upcoming elections through facilitating interactions with racially/ethnically diverse students would help high school students of all racial/ethnic groups be more prepared, motivate them to think critically about political issues, and increase their civic engagement, for example, inspiring them to participate in local volunteer activities and vote in upcoming elections.

Although, historically, young adults have voted at lower rates than older adults, the 2020 presidential election, whose turnout set a record, might prove to be a turning point in this pattern. Because voting tends to be a habitual behavior, many experts expect that the high young adult turnout rate in the 2020 election will continue. Indeed, the turnout in the 2022 midterm elections was higher than expected, and much higher than the 2014 turnout, although lower than the 2018 midterm turnout (the highest turnout for midterm elections in the 21st century). We are currently less than 6 months away from the 2024 presidential election. By all indicators, this election will be as close as the 2020 race, with the outcome determined by voter turnout in a few key battleground states. Young adults’ political participation in these states will likely play a pivotal role. We hope the findings reported in this article will reveal concrete actions that can maintain or increase voter turnout among young adults in future presidential elections.
